# Effect of Chemical Activation on Surface Properties of Poly(tetrafluoroethylene-co-hexafluoropropylene) Film

**DOI:** 10.3390/polym14214606

**Published:** 2022-10-30

**Authors:** Xuelei Li, Li Zhang, Hu Wang, Yongqing Zhao

**Affiliations:** 1 Science and Technology on Vacuum Technology and Physics Laboratory, Lanzhou Institute of Physics, Lanzhou 730000, China; 2 College of Chemistry and Chemical Engineering, Lanzhou University, Lanzhou 730000, China

**Keywords:** poly(tetrafluoroethylene-co-hexafluoropropylene), film surface, chemical activation, sodium naphthalene solution

## Abstract

Due to their low surface energy, poly(tetrafluoroethylene-co-hexafluoropropylene) (FEP) films must be treated by chemical or physical activation methods before using. Among these activation strategies, using sodium naphthalene solution is a popular one. However, the effect of this strategy’s chemical activation conditions on the surface properties of the FEP film is rarely discussed. In this study, FEP films were chemically activated by the sodium naphthalene solution with adjusting concentration, solvent, and activation time. With increasing concentration and activation time, many granular substances appeared on the surface of the FEP film. When tetrahydrofuran was used as a solvent, the color of the film gradually turned brown; when 1,3-dimethyl-2-imidazolidinone was chosen as the solvent, the color change was not very significant. The contact angle was significantly reduced from 112° before activation to 26° after activation, and the surface energy was greatly enhanced from 34 mN m^−1^ before activation to 66 mN m^−1^ after activation. In addition, compared with the FEP samples treated by Ar plasma, the sodium naphthalene system showed a stronger activation ability. Activated FEP films that suffered from the Ar plasma treatment could still maintain a higher energy surface than that of the pristine FEP.

## 1. Introduction

Poly(tetrafluoroethylene-co-hexafluoropropylene) (FEP) is a random polymer composed of tetrafluoroethylene and hexafluoropropylene. FEP has excellent high-temperature resistance, radiation resistance, corrosion resistance, and electrical properties, so it is widely used in the aerospace, petroleum, electronic communication, and chemical industries, as well as in medical treatment, textiles and other fields [1,2,3,4,5,6,7,8]. However, due to the existence of fluorine atoms in the molecular structure of FEP, its surface free energy is extremely low, resulting in a “low-energy surface” with no adhesiveness. This feature greatly limits the application of FEP in many fields, such as depositing functional films on its surface and compounding and bonding it with other polymer materials. Evidently, in these application scenarios, the surface of FEP must be activated to improve its surface energy and then the interface bonding force between FEP and other materials is enhanced [9,10,11,12,13]. At present, the methods of FEP surface activation treatment mainly include the following: chemical reduction method (sodium naphthalene solution, magnesium containing liquid ammonia solution, etc.), plasma treatment method, irradiation method (light and electron irradiation), silicic acid modification method, ion implantation method, and mechanochemical treatment method. For example, Li et al. utilized ^60^Co to radiate the FEP film and then easily grafted styrene onto the FEP substrate [1]. Ellinas et al. used a plasma micro-nanotexturing technology to tune the wetting and friction properties of FEP [2]. Clearly, these methods have their own advantages and disadvantages. For example, plasma treatment and irradiation methods usually require expensive and complex equipment, while chemical methods have potential environmental pollution problems [14,15,16,17,18,19,20,21,22,23,24]. Among them, the sodium naphthalene solution method has more merits, such as a simple process, low cost and good activation effect, and thus it is widely used. As early as 1956, Benderly et al. discovered that sodium naphthalene solution could blacken the surface of a Teflon stirrer in organic reactions and began research on the activation of polyfluorocarbons by this solution system. Through further research, they found that sodium naphthalene solution has many advantages in treating FEP and ordinary Teflon with complex shapes. For example, the solution can be stored and intermittently used without an exhaust device or inert atmosphere, and the activated sample does not need high-temperature treatment. The binding strength of the treated sample is almost the same as that of other activation methods (such as irradiation grafting and corona bombardment) [21]. McCarthy et al. studied the FEP surface layer activated by sodium naphthalene solution. They proposed that the surface layer was unsaturated and air-sensitive. In addition to C=C and C≡C, it also contains alcohol, carbonyl (possibly ketone), and aliphatic C-H bonds. The surface layer can be further modified by borohydride and subsequent oxidation to prepare a surface mainly containing hydroxyl groups [16]. In the reported study, fruitful research was carried out on the effect and mechanism of the activation of FEP by sodium naphthalene solution, but so far, there are still few public reports on the factors affecting the activation effect of sodium naphthalene solution. In this study, the surface of FEP film was activated by sodium naphthalene solution, and the effects of the activation conditions (concentration, treatment time and solvent) on its surface micromorphology, especially its chemical composition and surface energy, were further discussed. The goal was to provide a more comprehensive and rich understanding of the activation of FEP by sodium naphthalene solution.

## 2. Materials and Methods

### 2.1. Materials

Tetrahydrofuran (THF), 1,3-dimethyl-2-imidazolinone (DMI), refined naphthalene, hydrochloric acid, absolute ethanol, acetone, and diiodomethane were purchased from Beijing Innochem Science & Technology Co., Ltd (Beijing, China). All the reagents were used as received.

### 2.2. Preparation of Sodium Naphthalene Solution

Taking the preparation of 0.1 M sodium naphthalene solution as an example, 100 mL of THF or DMI was poured into a 250 mL round-bottom flask and then 1.28 g of refined naphthalene was added, which was completely dissolved by electromagnetic stirring. Next, 0.23 g of metal sodium was cut into small pieces and added into the above solution in batches. The reaction temperature was controlled at 5–15 °C until the solution had fully reacted and turned dark green, thus obtaining sodium naphthalene solution. The preparation method of other concentration sodium naphthalene solutions was the same, despite different feeding amounts of metal sodium and refined naphthalene.

### 2.3. FEP Surface Activation

Commercially available FEP film (DuPont, Wilmington, NC, USA) was used. Before chemical activation, the FEP film was soaked and cleaned with acetone to ensure the surface activation effect. The cleaned FEP film was immersed in a certain concentration of sodium naphthalene solution and left standing for a certain time. After taking it out, the FEP film was washed with dilute hydrochloric acid, distilled water, and absolute ethanol several times and then air-dried. After surface activation, the FEP film sample was labeled FEP-X-Y-Z, where X represents the solvent (THF or DMI), Y represents the concentration of sodium naphthalene solution, and Z represents the activation treatment time.

For comparative study, the FEP surface was also physically activated by Ar plasma. The FEP film was placed in the vacuum chamber of the magnetron sputtering coating equipment (1200RTR of Shengboer Photoelectric Technology Co., Ltd., Zhongshan, China) and vacuumized before being activated by ion beam. The vacuum chamber pressure was 0.15 Pa, the argon flow rate was 100 sccm, the ion source power was 1200 W and the tape speed of continuous winding was 0.3 m/min. The FEP film sample treated with Ar plasma was labeled as FEP-P. 

### 2.4. Characterization

A scanning electron microscope (SEM, Hitachi S4800, Tokyo, Japan) was used to characterize the micromorphology of the FEP film samples before and after chemical activation. The chemical composition of the FEP film surface before and after chemical activation was analyzed by X-ray photoelectron spectroscopy (XPS, Shimadzu Axis Supra, Kyoto, Japan) with a monochromatic Al Kα source, and the deconvolution and background treatment for the XPS spectra were carried out by the XPS Peak Fit software. A water drop angle (contact angle) tester (Baoda Instrument BT-200S, Dongguan, China) was used to measure the contact angle of the FEP film with water and diiodomethane before and after chemical activation. The surface energy was calculated according to the following equation:

(1) $$\gamma_{S}=\gamma_{S}^{d}+\gamma_{S}^{p}$$

(2) $$\left(1+\cos\theta \right)\gamma_{L}=2{\left(\gamma_{S}^{d}\gamma_{L}^{d}\right)}^{1/2}+2{\left(\gamma_{S}^{p}\gamma_{L}^{p}\right)}^{1/2}$$

where *γ_s_* (mN m^−1^), *γ_s_^d^* (mN m^−1^), *γ_s_^p^* (mN m^−1^), *γ_L_* (mN m^−1^), *γ_L_^d^* (mN m^−1^), *γ_L_^p^* (mN m^−1^), *γ_s_^d^* (mN m^−1^), *γ_L_^p^* (mN m^−1^), and *θ* (degree) is surface energy of the FEP film, dispersive component of *γ_s_*, polar component of *γ_s_*, surface energy of water/diiodomethane, dispersive component of *γ_L_*, polar component of *γ_L_*, and contact angle, respectively. The contact angle measurement and surface free energy calculation were performed by the contact angle measuring instrument software (SDC-100S).

## 3. Results and Discussion

### 3.1. Surface Morphology of FEP Films

**Figure 1:**
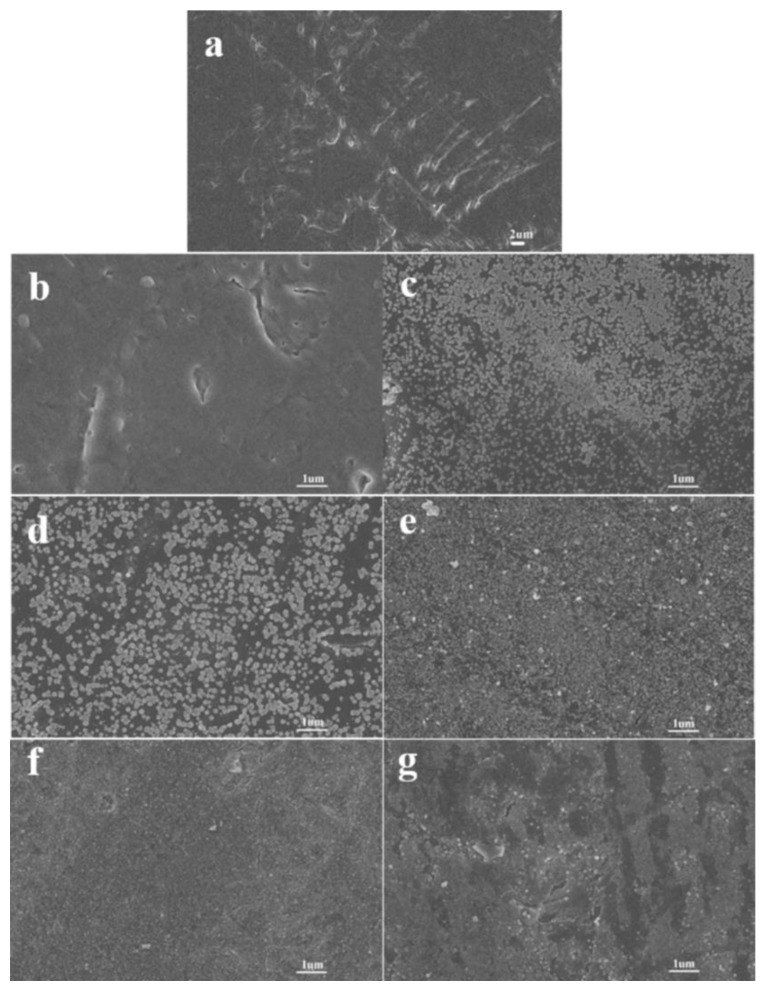
SEM images of the surface of FEP films activated by sodium naphthalene-THF solution with different concentrations and activation times: (**a**) FEP, (**b**) FEP-THF-0.1 M-10 s, (**c**) FEP-THF-0.1 M-30 s, (**d**) FEP-THF-0.2 M-10 s, (**e**) FEP-THF-0.2 M-30 s, (**f**) FEP-THF-0.3 M-10 s, (**g**) FEP-THF-0.3 M-30 s.

The surface micromorphology of FEP films activated with different concentrations of sodium naphthalene-THF solution for different durations is shown in Figure 1. It is clear that the surface of the pristine FEP is relatively smooth (as shown in Figure 1a), while the surface of the activated FEP is rough. When the concentration of sodium naphthalene-THF solution and the activation time increased, the surface of FEP became rougher, and a large number of granular substances appeared (as shown in Figure 1b–g). The reported activation mechanism for the FEP activated by sodium naphthalene-THF solution [16] shows that sodium-positive ions originating from the reaction between sodium and naphthalene can cut the C-F bond, and derivatives of naphthalene may be formed by washing the activated FEP surface with diluted hydrochloric acid and water.

**Figure 2:**
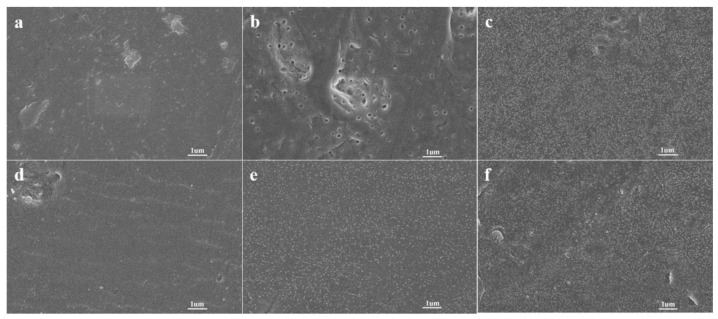
SEM images of the surface of FEP films activated by sodium naphthalene-DMI solution with different concentrations and activation times: (**a**) FEP-DMI-0.7 M-5 min, (**b**) FEP-DMI-0.7 M-15 min, (**c**) FEP-DMI-0.7 M-30 min, (**d**) FEP-DMI-0.9 M-5 min, (**e**) FEP-DMI-0.9 M-15 min, (**f**) FEP-DMI-0.9 M-30 min.

**Figure 3:**
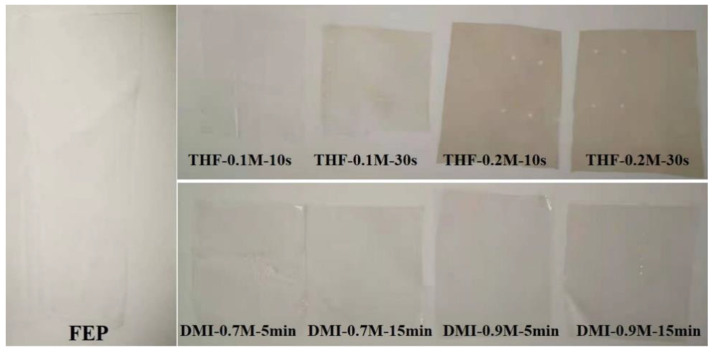
Photographs of the FEP films activated by sodium naphthalene solution.

As shown in Figure 2, when the sodium naphthalene-DMI solution was used to activate the FEP film, the phenomenon was similar to that of the sodium naphthalene-THF system, but the sodium naphthalene-DMI system needed a higher concentration and longer activation time to achieve the similar activation effect. In addition, it is worth noting that, as shown in Figure 3, when THF was used as the solvent the color of the FEP film activated by sodium naphthalene solution turned brown; this color gradually deepened with the increase in the concentration of sodium naphthalene-THF solution and the extension of the activation time. However, when DMI was used as the solvent, the color change was not significant. Clearly, the change of solvent has a significant effect on the activation of FEP by sodium naphthalene solution.

### 3.2. Surface Chemical Composition of FEP Films

The chemical composition of the FEP film before and after chemical activation was analyzed by XPS. As shown in Table 1, before chemical activation the surface of the FEP film was mainly composed of C and F elements and contained a small amount of O element that may have been caused by impurities and adsorbed water. The F/C ratio is 2.00 and the O/C ratio is only 0.04, which is consistent with the reported results in the previous studies [18,20]. After the FEP film was activated by sodium naphthalene solution, the amount of F element was significantly reduced, while the amounts of C and O elements were dramatically increased. This indicates that the sodium naphthalene solution can effectively cut off the C-F bond, introduce O element, and increase the surface activity. When the sodium naphthalene-THF system’s concentration increased to 0.2 M, only 10 s of activation was required, the F/C ratio decreased sharply to 0.14, and the O/C ratio increased to 0.29. However, after further increasing the concentration of sodium naphthalene solution (such as to 0.3 M) and increasing the activation time (such as to 30 s), the amounts of C and F elements could not change much; that is, the activation effect could not change significantly. Moreover, the sodium naphthalene-DMI system shows a similar activation tendency to the sodium naphthalene-THF system, but it required a higher concentration and longer activation time. For example, when the concentration was increased to 0.9 M and the activation time was 5 min, the F/C and O/C ratios of the obtained sample FEP-DMI-0.9 M-5 min reached to those of FEP-THF-0.2 M-10 s. In addition, compared with FEP-P samples treated by Ar plasma, the FEP samples activated by the sodium naphthalene system possessed a lower F/C ratio and a higher O/C ratio, namely, the sodium naphthalene system showed a stronger activation ability than that of the Ar plasma treatment.

**Figure 4:**
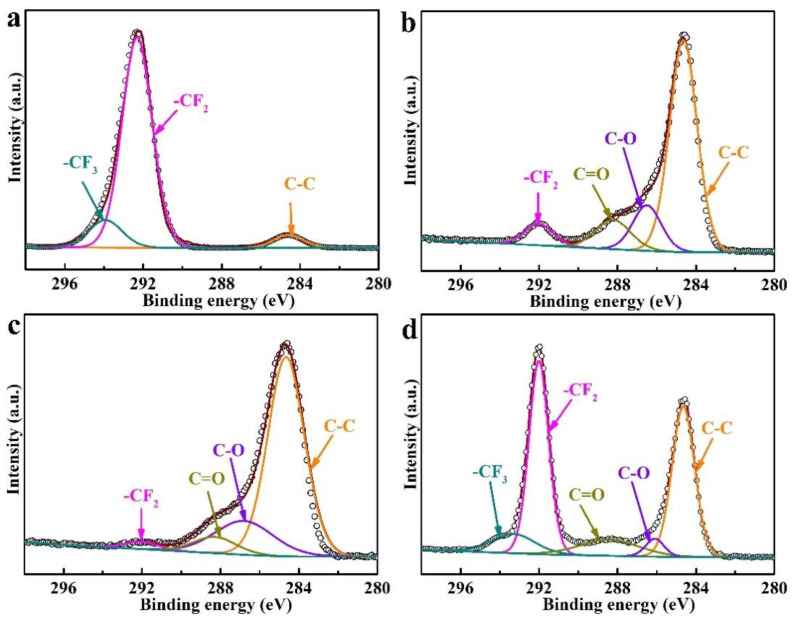
XPS C1s spectra of FEP films activated under different conditions: (**a**) FEP, (**b**) FEP-THF-0.3 M-30 s, (**c**) FEP-DMI-0.9 M-30 min, (**d**) FEP-P.

In order to further confirm the functional groups on the surface of the FEP films activated by different chemical activation conditions, the C1s and O1s fine spectra of relevant samples were subjected to peak splitting. In the C1s spectra (as shown in Figure 4), peaks with binding energies of ~284.7, ~286.5, ~288.3, ~292.2, and ~293.6 eV correspond to C-C, C-O, C=O, -CF_2,_ and -CF_3_ groups [20,25,26,27], respectively. Additionally, it can be clearly observed that the surface of the pristine FEP was mainly composed of -CF_2_ and -CF_3_ groups (as shown in Figure 4a). However, after chemical activation with sodium naphthalene system, for example, the peak intensity of the -CF_2_ group on the surface of FEP-THF-0.3 M-30 s (Figure 4b) and FEP-DMI-0.9 M-30 min (Figure 4c) sharply decreased. The -CF_3_ group could not be detected, while the peak intensity of the C-C bond increased significantly, and C-O and C=O groups appeared. This further proves that the sodium naphthalene system can effectively cut the C-F bond of the -CF_2_ and -CF_3_ groups, and successfully introduce oxygen-containing groups. On the other hand, although oxygen-containing groups also could be introduced into the FEP film by the Ar plasma treatment, the peak intensity of the -CF_2_ and -CF_3_ groups still maintained a high level (Figure 4d).

**Figure 5:**
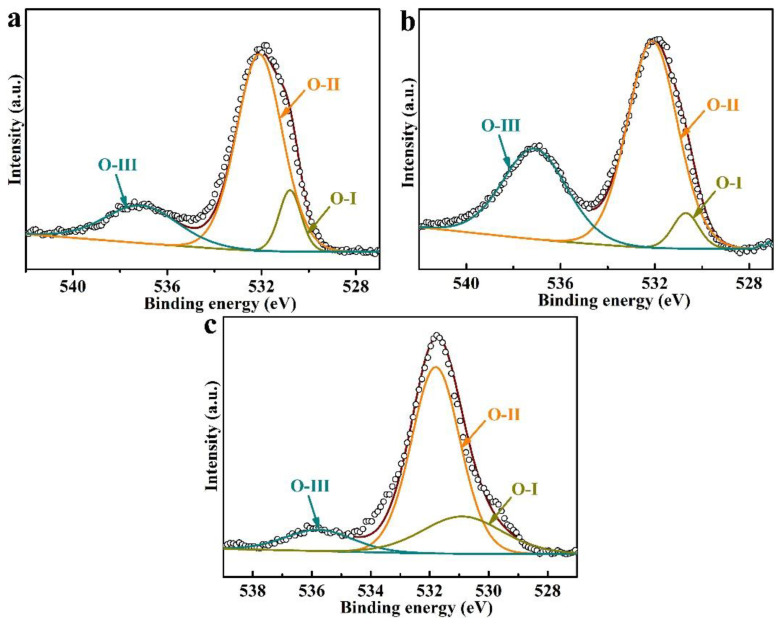
XPS O1s spectra of FEP films activated under different conditions: (**a**) FEP-THF-0.3 M-30 s, (**b**) FEP-DMI-0.9 M-30 min, (**c**) FEP-P.

In addition, through analyzing the O1s spectra (as shown in Figure 5), it can be seen that there are three types of oxygen-containing groups on the surface of the activated FEP films, namely O-I (~531.0 eV, C=O quinone groups), O-II (~532.1 eV, C-OH phenols and C-O-C ether groups), and O-III (~536.0 eV, adsorbed oxygen and COOH groups) [28,29], of which the proportion of O-II oxygen-containing groups is the highest. It is also noted that the type of oxygen-containing groups of the activated FEP films is similar despite different activation strategies (the sodium naphthalene system and Ar plasma treatment).

### 3.3. Contact Angle and Surface Energy of FEP Films

**Figure 6:**
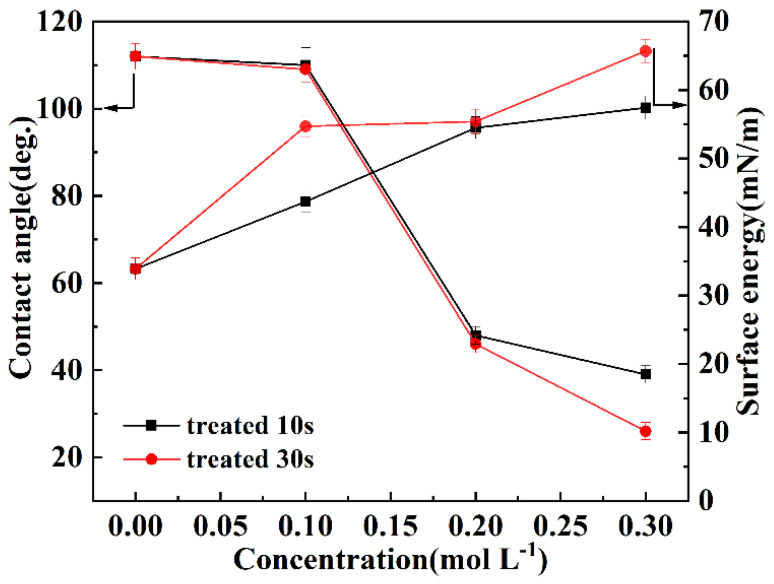
The variation trend of the contact angle and surface energy of FEP films with the concentration of sodium naphthalene-THF solution.

As shown in Figure 6, when the surface of FEP film was activated in the sodium naphthalene-THF solution, the contact angle dramatically decreased as the concentration of sodium naphthalene-THF solution increased. As the concentration was higher than 0.1 mol L^−1^, the reduction in the contact angle was particularly evident; it reduced from 112° before activation to 26° after activation. Namely, before and after activation the FEP surface showed a change from a hydrophobic to hydrophilic property, which is due to the fact that fluorine-containing groups (-CF_2_ and -CF_3_ groups) of the pristine FEP were partly replaced by oxygen-containing groups (especially C-OH phenols and C-O-C ether groups) after activation. On the other hand, with the increase in the concentration of sodium naphthalene-THF solution, the surface energy of the FEP film showed a tendency to increase: it increased from 34 mN m^−1^ before activation to 66 mN m^−1^ after activation. This result further suggests that the sodium naphthalene-THF solution can effectively activate the FEP film, and that the degree of activation can be controlled by adjusting the concentration of the sodium naphthalene-THF solution, which is also consistent with the previous XPS analysis results. In addition, it also can be observed that the effect of activation time on the contact angle and surface energy is less than that of the concentration of the sodium naphthalene-THF solution. At the same concentration of sodium naphthalene-THF solution, increasing the activation time could not significantly decrease the contact angle and increase surface energy.

**Figure 7:**
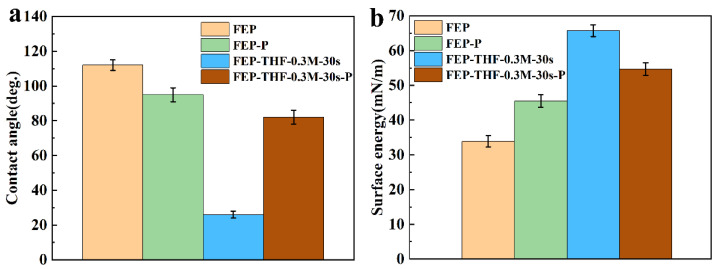
Comparison diagram of the contact angle and surface energy of FEP films under different activation conditions of the sodium naphthalene-THF system: (**a**) contact angle, (**b**) surface energy.

**Figure 8:**
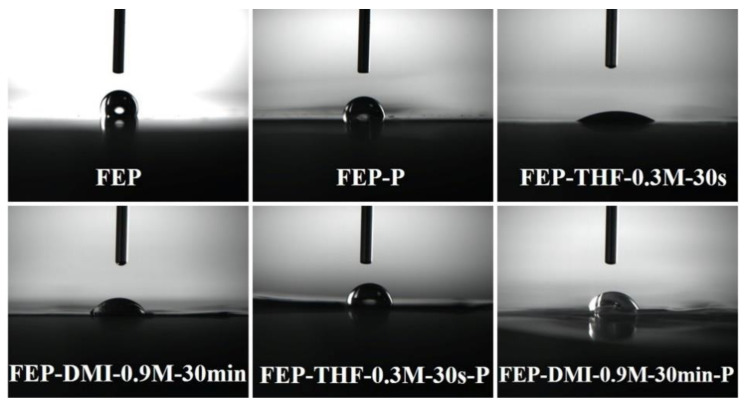
Photographs of the contact angle test for the FEP film with water under different activation conditions.

To further understand the effects of activation conditions on the surface properties of the FEP films, the contact angle and surface energy of the FEP under different activation conditions were compared, as shown in Figure 7 and Figure 8. Evidently, compared with the Ar plasma activation, sodium naphthalene-THF solution has a stronger activation effect. For example, the contact angle of the FEP-P sample was 95°, while the contact angle of FEP-THF-0.3 M-30 s was only 26°. When the surface of the FEP-THF-0.3 M-30 s sample was subjected to the Ar plasma treatment under the same conditions as the FEP-P sample, the contact angle of FEP-THF-0.3 M-30 s-P increased to 82°, but it was still smaller than that of FEP (112°) and FEP-P. On the other hand, the surface energy of the FEP-THF-0.3 M-30 s sample (66 mN m^−1^) is the highest among the four samples. And that of FEP-THF-0.3 M-30 s-P could reach to 55 mN m^−1^, which is still higher than that of the FEP (34 mN m^−1^) and FEP-P (46 mN m^−1^). These results further suggest that the FEP surface activated by sodium naphthalene-THF solution has sufficient oxygen-containing groups, even after being treated by Ar plasma again, which ensured good surface hydrophilicity of the activated FEP films.

**Figure 9:**
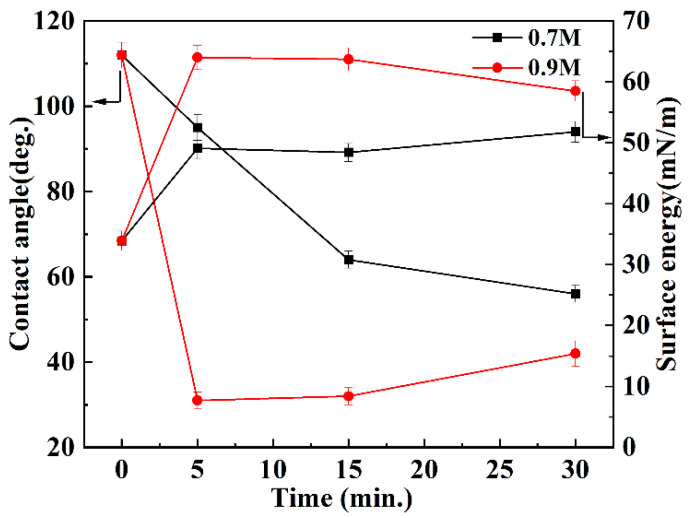
The variation trend of the contact angle and surface energy of FEP films with the activation time in the sodium naphthalene-DMI solution.

The effects of activation conditions of sodium naphthalene-DMI system on the contact angle and surface energy of the FEP films was also investigated. As shown in Figure 9, when the FEP film was activated in the sodium naphthalene-DMI solution, its contact angle shows a decreasing tendency with the increase in activation time; the angle reduced from 112° before activation to 31° after activation. The surface energy first increased with the activation time, but when the activation time surpassed 5 min it could not vary significantly, and the contact angle decreased slowly. The surface energy increased from 34 mN m^−1^ before activation to 64 mN m^−1^ after activation. At the same activation time, the contact angle decreased, and the surface energy increased as the concentration of sodium naphthalene-DMI solution was increased. It can be seen that, consistent with the above XPS analysis results, sodium naphthalene-DMI solution can effectively activate FEP films, and the degree of activation can be controlled by adjusting the concentration and activation time of the sodium naphthalene-DMI system.

**Figure 10:**
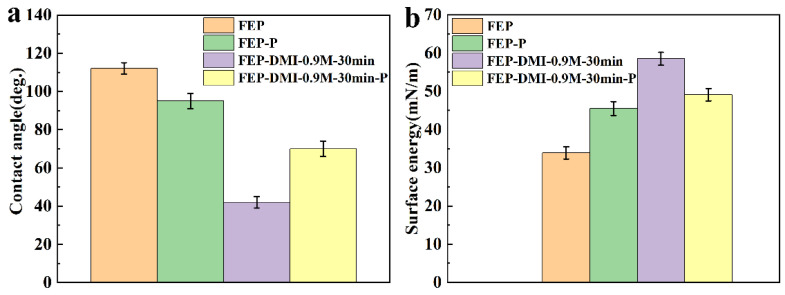
Comparison diagram of the contact angle and surface energy of FEP films under different activation conditions of the sodium naphthalene-DMI system: (**a**) contact angle, (**b**) surface energy.

The surface contact angle and surface energy of the FEP films under different activation conditions were also compared in the sodium naphthalene-DMI system. As shown in Figure 8 and Figure 10, compared with the Ar plasma activation strategy, the sodium naphthalene-DMI solution has a stronger activation effect. For example, the contact angle of FEP-P was 95°, but that of FEP-DMI-0.9 M-30 min decreased to 42°. Moreover, if the surface of FEP-DMI-0.9 M-30 min sample was treated with the Ar plasma under the same conditions for FEP-P, its contact angle would increase to 70°, but it was still smaller than that of FEP (112°) and FEP-P (95°). Meanwhile, the FEP-DMI-0.9 M-30 min sample had the highest surface energy (59 mN m^−1^), and the surface energy of FEP-DMI-0.9 M-30 min-P (49 mN m^−1^) was higher than that of FEP (34 mN m^−1^) and FEP-P (46 mN m^−1^). These results also show that FEP films activated by a sodium naphthalene-DMI solution can maintain a high surface energy, even after being treated by Ar plasma again, which is due to a large number of oxygen-containing groups on its surface.

## 4. Conclusions

In summary, the sodium naphthalene system can effectively activate the surface of FEP films. The activation system can easily cut the C-F bond in -CF_2_ and -CF_3_ groups of FEP films. After acid and water washing, oxygen-containing groups, such as hydroxyl and carboxyl groups, can be introduced onto the surface of FEP films. The variation in concentration, solvent, and activation time of the activation system can lead to different surface properties (such as functional groups, contact angle, and surface energy) of the FEP films. In particular, the solvent has an important influence on its activation ability. Compared with the sodium naphthalene-DMI system, the sodium naphthalene-THF system has a stronger activation ability. After being treated with the sodium naphthalene-THF solution with a lower concentration and shorter activation time, a FEP film changes color, its surface contact angle is significantly reduced, and its surface energy is greatly increased. Clearly, the degree of activation for the FEP films can be easily controlled by adjusting the activation conditions of the sodium naphthalene system. It is worth noting that the chemically activated FEP films, which suffered from the Ar plasma treatment, can still maintain a higher surface energy than that of the pristine FEP. This will promote the application potential of the chemically activated FEP films in the field of vacuum coating.

## Figures and Tables

**Table 1:** XPS element content of the surface of FEP activated under different conditions.

Samples	C (at.%)	F (at.%)	O (at.%)	F/C	O/C
FEP	32.85	65.76	1.40	2.00	0.04
FEP-P	46.44	47.53	6.03	1.02	0.13
FEP-THF-0.1 M-10 s	41.22	56.32	2.46	1.37	0.06
FEP-THF-0.2 M-10 s	69.51	10.01	20.48	0.14	0.29
FEP-THF-0.3 M-10 s	62.06	18.04	19.90	0.29	0.32
FEP-THF-0.3 M-30 s	65.89	14.14	19.95	0.21	0.30
FEP-DMI-0.7 M-5 min	50.28	38.70	11.01	0.77	0.22
FEP-DMI-0.9 M-5 min	69.58	11.50	18.93	0.17	0.27
FEP-DMI-0.9 M-30 min	65.11	17.72	17.18	0.27	0.26

## Data Availability

Not applicable.
